# Supplementary material for: The Capacity of APOB-Depleted Plasma in Inducing ATP-Binding Cassette A1/G1-Mediated Macrophage Cholesterol Efflux—But Not Gut Microbial-Derived Metabolites—Is Independently Associated with Mortality in Patients with ST-Segment Elevation Myocardial Infarction
Source: Biomedicines. 2021 Sep 27;9(10):1336. doi: 10.3390/biomedicines9101336 (PMC8533386; doi:10.3390/biomedicines9101336)
Supplement: Supplementary file 1 [file biomedicines-09-01336-s001.zip › biomedicines-1356732-supplementary.pdf]

biomedicines

The Capacity of APOB-Depleted Plasma in Inducing ATP-Binding Cassette A1/G1-Mediated Macrophage Cholesterol Efflux—But not Gut Microbial-Derived Metabolites—Is Independently Associated with Mortality in Patients with ST-Segment Elevation Myocardial Infarction

Supplementary materials

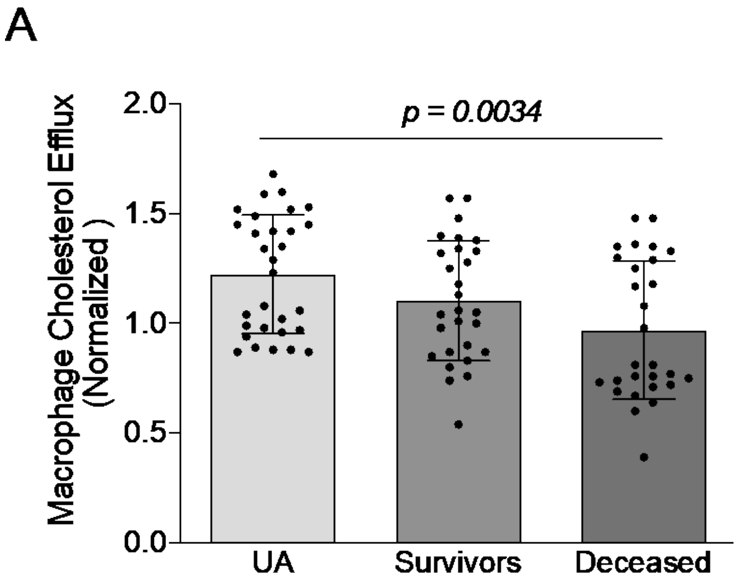

Figure S1. Macrophage cholesterol efflux after activation of macrophage ABCA1 by cAMP (ANOVA p value < 0.0001) (B). Values are represented as mean ± SD in UA, STEMI survivors and deceased STEMI.

Table S1. Analysis of ANCOVA of Baseline (a) and ABCA1-mediated (b) macrophage cholesterol efflux at 24 hours.

(a)

**Source**	**SS**	**df**	**Mean Square**	**F**	**p Value**
Corrected Model	1.793*	5	0.359	4.805	0.001
Intercept	1.930	1	1.930	25.865	0.000
HDL-C	0.913	1	0.913	12.242	0.001
eGFR	0.045	1	0.045	0.609	0.438
TMAO	0.013	1	0.013	0.175	0.677
Death	0.245	2	0.122	1.640	0.200
Error	5.895	79	0.075		
Total	110.274	85			
Corrected Total	7.688	84			
*R Squared = 0.255 (Adjusted R Squared = 0.214)					

(b)

**Source**	**SS**	**Df**	**Mean Square**	**F**	**p Value**
Corrected Model	0.769*	5	0.154	6.219	0.000
Intercept	2.286	1	2.286	92.408	0.000
HDL-C	0.429	1	0.429	17.345	0.000
eGFR	0.000	1	0.000	0.015	0.904
TMAO	0.058	1	0.058	2.355	0.128
Death	0.108	2	0.054	2.191	0.118
Error	2.251	91	0.025		
Total	106.402	97			
Corrected Total	3.020	96			
*R Squared = 0.233 (Adjusted R Squared = 0.185)					
